# Steel Plates Versus Hybrid CFRP/Steel Stirrups for Strengthening of Shear-Deficient Concrete Wide Beams Supporting Columns

**DOI:** 10.3390/polym17212857

**Published:** 2025-10-26

**Authors:** Omar Al-Hamed, Aref Abadel, Tarek Almusallam, Hussein Elsanadedy, Husain Abbas, Yousef Al-Salloum

**Affiliations:** Chair of Research and Studies in Strengthening and Rehabilitation of Structures, Department of Civil Engineering, College of Engineering, King Saud University, P.O. Box 800, Riyadh 11421, Saudi Arabia; 442106952@student.ksu.edu.sa (O.A.-H.); aabadel@ksu.edu.sa (A.A.); musallam@ksu.edu.sa (T.A.); helsanadedy@ksu.edu.sa (H.E.); habbas@ksu.edu.sa (H.A.)

**Keywords:** shear strengthening, RC wide beams, planted columns, steel plates, planted steel stirrups, testing, analytical modeling

## Abstract

Joist floor systems are usually employed in buildings of the Middle Eastern regions. These systems usually have reinforced concrete (RC) wide beams, which in many cases have planted columns in non-seismic regions due to architectural requirements. Changes in building use can increase the loads on these columns, which may increase the shear demand of beams to a level that may exceed their capacity. Consequently, upgrading of such wide beams against shear is crucial. This study investigates two strengthening techniques to enhance the shear performance of RC wide beams with planted columns through experimental testing and analytical evaluation. Four half-scale specimens were tested: two unstrengthened beams (one code-compliant and one shear-deficient) and two strengthened beams, using either externally bonded steel plates or a combination of CFRP U-wraps with planted steel U-stirrups. The performance of the retrofitting schemes was assessed based on failure modes and load-deflection responses. The second strengthening scheme improved the shear resistance of wide beams by 82% compared to the control specimen. Additionally, the shear capacity of the tested beams was analytically predicted, and the results were compared with the test findings, providing insights into the effectiveness of both strengthening methods.

## 1. Introduction

Reinforced concrete (RC) wide beams are popularly used in joist floor systems to increase the space of the structure and decrease the total height in residential buildings, commercial buildings and garages. These wide beams are commonly employed as economical transfer members in cases where minimizing the overall depth is required [1]. Wide beams are generally identified by their section dimensions, where the ratio of width to depth is larger than 2 [2,3], and the depth of the wide beam is typically less than 350 mm [4]. The ACI code 318-2019 [5] defines beams as wide if their depth is 250 mm or less, therefore exempting them from the need for a minimum amount of shear stirrups. Nevertheless, the Saudi Building Code SBC 304-18 [6] lacks a clear definition of wide beams in this context and does not prescribe minimum provisions for shear reinforcement design.

Due to their lower depth-to-width ratio, wide beams exhibit distinct shear failure mechanisms, prompting several studies on the contribution of web reinforcement towards shear strength [7,8,9]. Soliman et al. [10] found that increasing the concrete’s strength positively affected the shear resistance, although the impact of additional compression reinforcement was minimal. An experimental investigation by Lubell et al. [11] examined how varying the transverse distribution of shear rebar legs in the beam’s width influences the behavior of thirteen wide RC beams. The results showed that increasing leg spacing reduced the shear rebar efficiency. Shuraim [12] investigated how various stirrup configurations affect the shear resistance of wide beams. The study tested 16 RC wide beams with various stirrup configurations and found that stirrup configurations with four legs demonstrated significantly greater effectiveness compared to those with two legs in enhancing shear capacity, even when the stirrup area was small.

In the same context, some studies in the literature concentrated on retrofitting for improving the shear resistance of wide beams. Steel plates are commonly employed to retrofit structural members or repair damaged ones by bonding them to surfaces using adhesive materials or bolts [13]. Abbas et al. [3] tested nine wide beams to assess the effectiveness of side-bonded steel plates. The findings indicated that the shear resistance and the shear cracking load are both enhanced by the presence of steel plates.

Moreover, fiber-reinforced polymers (FRP) have gained attention for their effectiveness in shear retrofitting of RC conventional beams [14], RC beams containing openings [15], deficient deep beams [16], deficient RC beam-column joints [17], and RC columns [18,19]. Abass and Hassan [20] studied the shear response of RC wide beams and the efficacy of carbon FRP (CFRP) in upgrading their shear capacity. The outcomes demonstrated that the peak loads for wide beams retrofitted with inclined and vertical CFRP sheets enhanced by 19.9% and 7.14%, respectively, compared to the control wide beam. Kim et al. [21] tested six wide concrete beams retrofitted with glass FRP (GFRP) plates. The transverse spacing of shear rebars was taken into consideration as a parameter. The results revealed that the shear behavior of the retrofitted wide beams improved with decreasing spacing of shear stirrups, up to a spacing that reached 1.16 times the effective depth, beyond which the performance declined.

The application of advanced materials, e.g., Strain-Hardening Cementitious Composites (SHCC), has been investigated for shear strengthening purposes. Research has shown that the use of SHCC jackets along the shear spans significantly improves shear resistance [22]. Furthermore, Moubarak et al. [23] used internal and external fasteners installed vertically or at an incline within the shear critical zones for retrofitting wide beams. The test outcome demonstrated that their method successfully enhanced the shear resistance of wide beams. Furthermore, when comparing the specimen shear resistance of inclined fasteners to that of vertical fasteners, there was no discernible difference.

Despite the considerable research on the shear behavior and strengthening of RC wide beams, limited studies have focused on beams supporting planted columns, where complex stress transfer and high shear concentrations occur near the column interface. Moreover, while several investigations have explored shear enhancement using either steel plates or FRP composites, comparative assessments of these two widely adopted strengthening schemes for wide beams under combined shear and column load effects are scarce. Also, the use of externally bonded CFRP U-wraps combined with planted steel U-stirrups has been introduced in the current study as a novel technique for shear enhancement of RC shallow beams. The existing literature has largely concentrated on conventional slender beams, leaving a gap in understanding how such retrofit systems perform in shear-deficient shallow, wide members. In this context, the present study investigates the effectiveness of two practical and contrasting strengthening techniques: (i) external steel plates, representing a conventional, cost-effective retrofit option, and (ii) a hybrid system comprising CFRP wraps and planted U-stirrups, for improving the shear performance of RC wide beams supporting planted columns. The use of beams with planted columns was adopted to replicate a common structural detail frequently used in the Middle East and other non-seismic regions, where secondary columns are often constructed on existing beams to support additional floors or architectural extensions.

## 2. Experimental Campaign

### 2.1. Test Specimens

**Figure 1:**
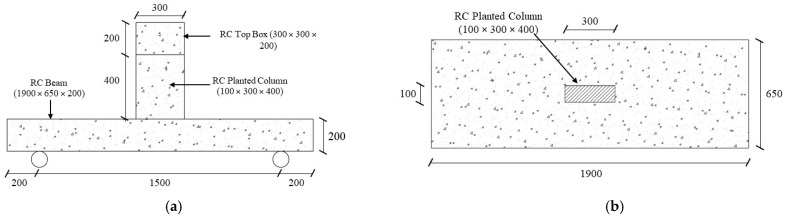
Dimensions of planted column specimens with RC wide beams. (**a**) Elevation; (**b**) Top view (Unit: mm).

In this research, four ½ scale RC wide beams (650 × 200 (deep) × 1900 mm) supporting columns were cast and subjected to three-point bending tests, as presented in Figure 1. To ensure consistency, a constant shear span-to-effective depth ratio (a/d) of 4.4 was maintained across all specimens. The chosen a/d ratio for three-point bending was driven by the study objective of simulating the practical condition of a wide beam that carries a column load applied through a relatively shallow shear span. The supports consisted of two steel rollers, each 60 mm in diameter. Table 1 provides the experimental details and specimen details.

**Figure 2:**
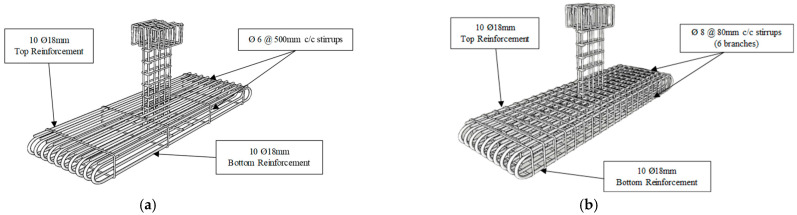
Reinforcement detailing of test specimens; (**a**) Control and strengthened specimens; (**b**) BS-ACI specimen.

As shown in Table 1, the control wide beam (BS-ACI) was designed based on the ACI 318-19 code [5] for normal beams to be sufficient in shear. However, the specimen BS-CON was intentionally designed to be deficient in shear. For the first strengthening scheme (in specimen BS-S1), steel plates were used, and CFRP sheet and steel U-stirrups were used in the second strengthening scheme (specimen BS-S2). All beams were reinforced with 10Ø18 mm longitudinal tension rebars arranged in a single bottom layer, which is corresponding to 2.3% of flexural reinforcement, and 10Ø18 mm top rebars were used as compression reinforcement of the wide beam, as shown in Figure 2.

### 2.2. Properties of Materials

The materials properties of concrete, steel reinforcements, and strengthening materials utilized in this work are presented in Table 2. Ready-mix concrete was employed to cast wide beam specimens with planted columns. Standard cylinders of concrete (150 × 300 mm) were cast and tested in accordance with the relevant ASTM standard [24] to determine concrete strength at 28 days and on the day of testing specimens. Tension tests were conducted on the steel rebars according to ASTM E8/E8M [25] to determine the properties listed in Table 2. Steel plates and CFRP sheets were utilized for strengthening schemes. Tension test coupons for steel plates were machined, cut, and tested in accordance with ASTM A370-17 [26]. An epoxy-based adhesive mortar [27] was used for bonding the steel plates to the RC wide beams, and the holes prepared for the installation of U-stirrups were filled using Sika AnchorFix^®^-2 [28]. The average properties of steel plates and CFRP sheets are shown in Table 2.

### 2.3. Properties of Materials

#### 2.3.1. Shear Strengthening Using Steel Plate (BS-S1)

**Figure 3:**
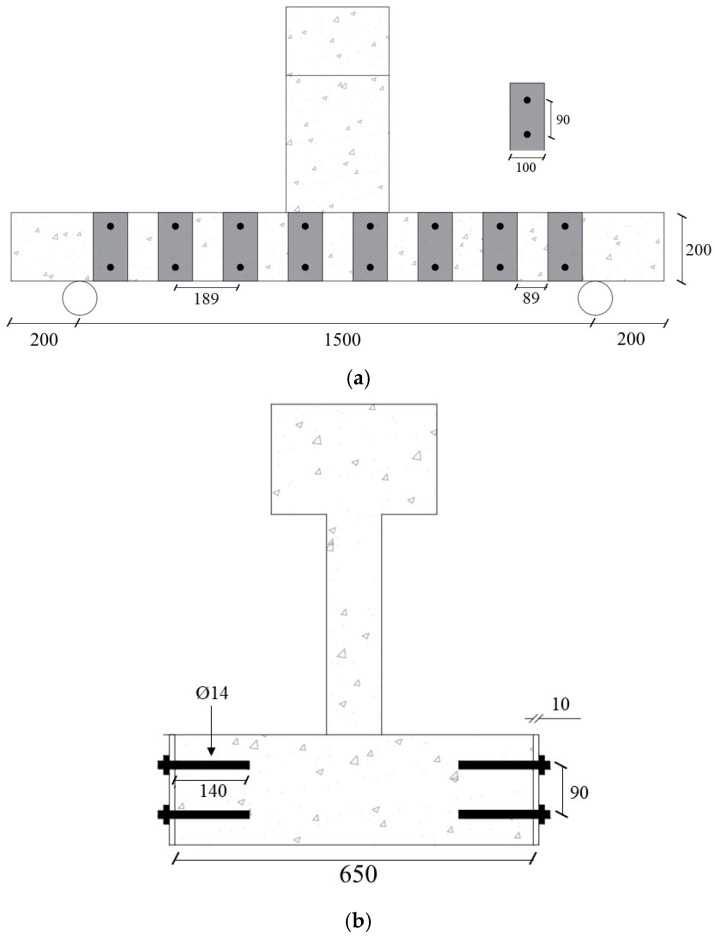

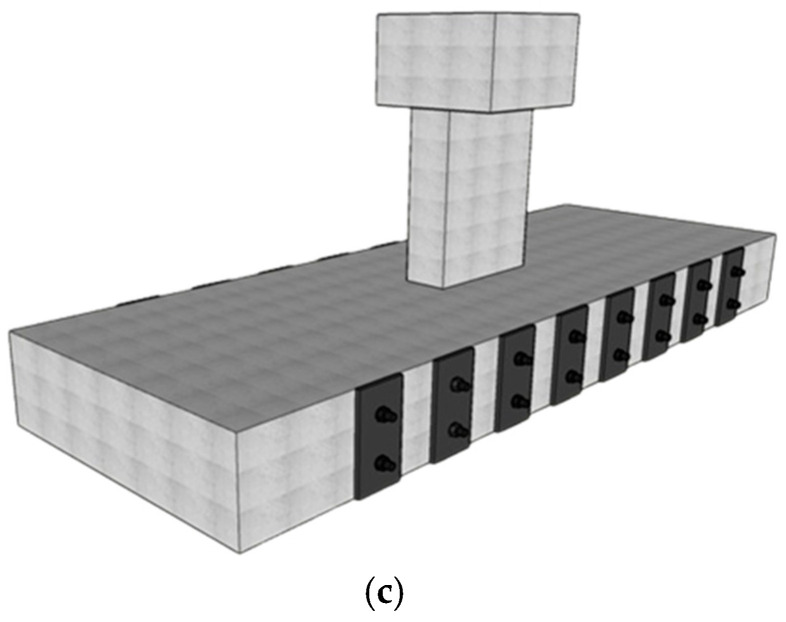
Strengthened specimen BS-S1 details: (**a**) Elevation; (**b**) Lateral view; (**c**) Isometric perspective (Unit: mm).

This strengthening scheme was implemented in specimen BS-S1 as shown in Figure 3. In this strengthening scheme, steel plates measuring (200 × 100 × 10 mm) were attached to both sides of the wide beam at a center-to-center spacing of 189 mm. The steel plates of 10 mm thickness were adhesively bonded (using Sikadur-31) to the lateral surfaces of the specimen, as shown in Figure 3. The plates were also tied to the wide beam using steel rods of 14 mm diameter. As these steel rods were in the middle of the steel plates, their center-to-center spacing was also 189 mm. The diameter of the holes for the threaded rods was 18 mm to allow for grouting using the adhesive mortar. After the adhesive mortar had cured, the high-strength rods were secured by tightening them with washers and nuts.

#### 2.3.2. Shear Strengthening Using CFRP Sheet with U-Stirrups (BS-S2)

**Figure 4:**
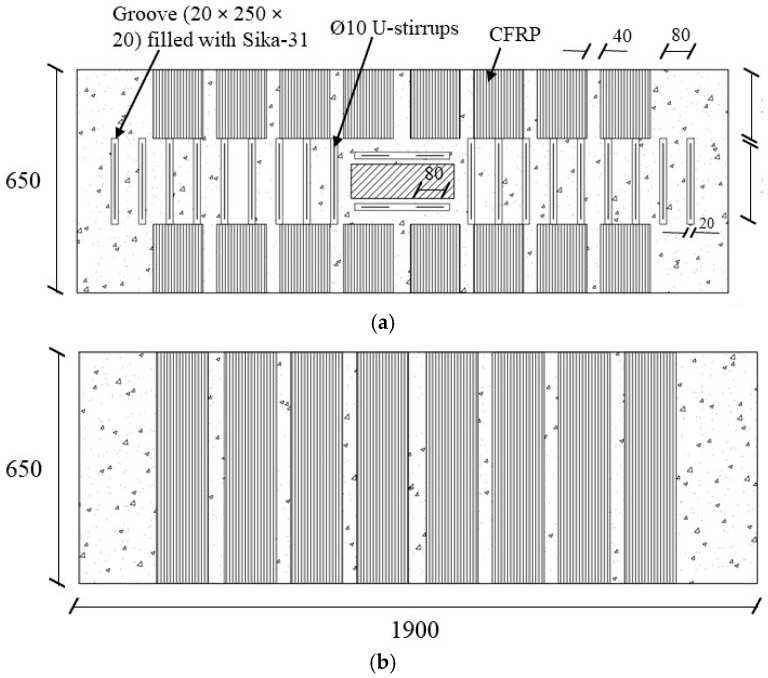

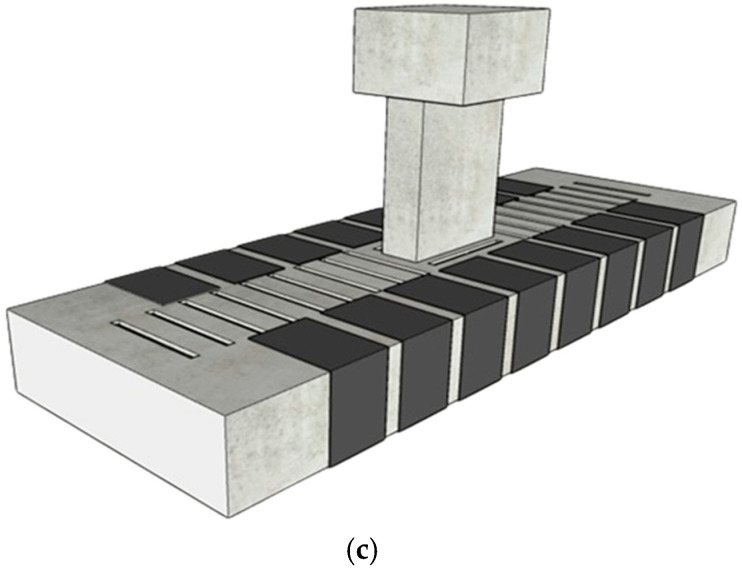
Strengthened specimen BS-S2 details: (**a**) Top view; (**b**) Bottom view; and (**c**) Isometric perspective (Unit: mm).

This strengthening scheme was implemented in specimen BS-S2 as depicted in Figure 4. This scheme contained a U-shaped CFRP sheet that was applied around the beam. The design of the strengthening scheme was according to the equations available in ACI 440.2R-17 [29], with an additional 200 mm of CFRP sheet extending from both sides of the top surface to prevent debonding. The outer surface of the wide beam was leveled to obtain a smooth concrete surface. CFRP sheets were bonded using the conventional wet-layup technique, which is commonly employed in FRP applications. Initially, a thin layer of adhesive resin was spread on the surface of the wide beam, after which the CFRP sheet was fully saturated with the resin. Care was taken to eliminate any air voids in the CFRP sheet when applying it to the wide beam surface. Additionally, 10 mm U-stirrups were incorporated into the beam from the top, with AnchorFix-2 [28] used to fill the drilled holes of the stirrups as shown in Figure 4. This comprehensive approach aimed to enhance the shear capacity of the wide beam, incorporating both CFRP sheets and steel U-stirrups for an effective strengthening solution.

### 2.4. Testing Configuration and Instrumentation

**Figure 5:**
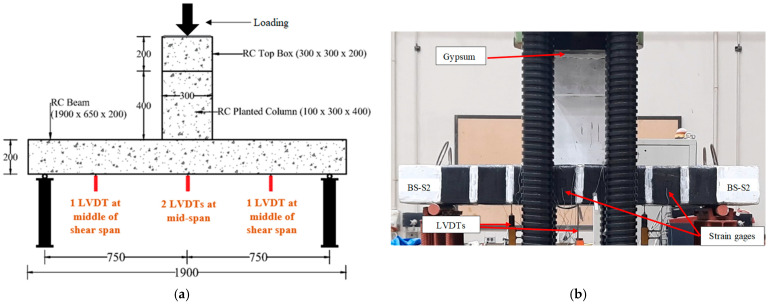
Setup for testing wide beam specimens (Unit: mm); (**a**) LVDT locations; (**b**) Specimen prepared for test.

The experimental setup prepared to test wide beams is illustrated in Figure 5. All wide beams were subjected to a three-point bending test configuration by a test machine of 2000 kN capacity. The planted column was subjected to a monotonically increasing load via a displacement control strategy, at a loading rate of 2 mm/min. The mid-span displacement was measured using a linear variable displacement transducer (LVDT), while strain gauges affixed to the wide beams recorded the strains in the rebars, steel plates, and CFRP sheet. It should be mentioned that to ensure a uniform pressure during testing, a layer of gypsum was placed on the cap of the planted column.

## 3. Test Results and Discussion

The test findings, including failure modes and load-deflection response, are discussed in the following.

### 3.1. Failure Modes

#### 3.1.1. Control Specimen BS-CON

**Figure 6:**
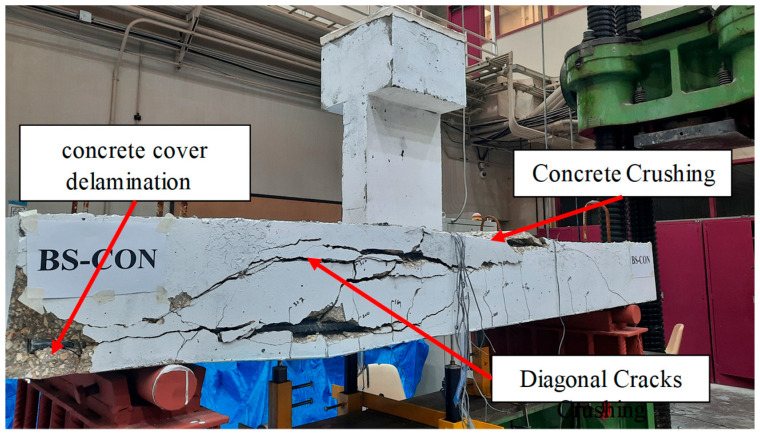
Final failed control specimen BS-CON.

The mode of failure of specimen BS-CON is depicted in Figure 6. The flexure cracks were first noticed just below the planted column at a load of 45 kN. With increasing loading, diagonal shear cracks were detected at 169 kN. Figure 6 clearly indicated that the diagonal crack that extended from the planted column to the supports largely affected the behavior of the wide beam up to failure at the load level of 330 kN. Subsequent to shear failure, crushing of concrete was noted in the compression zone of specimen BS-CON as illustrated in Figure 6.

#### 3.1.2. Control Specimen BS-ACI

**Figure 7:**
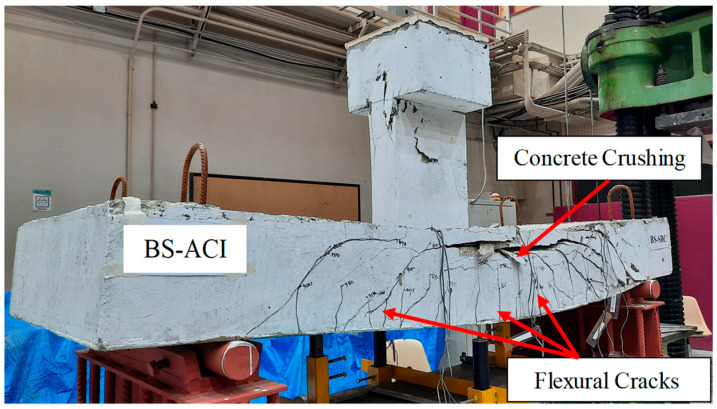
Final failed condition of BS-ACI.

The final mode of failure of BS-ACI is presented in Figure 7. The initial visible flexural crack was observed near mid-span under a load of 74 kN, while the first diagonal crack appeared when the load approached 295 kN, corresponding to approximately 46% of the ultimate load. The specimen failed due to the widening of flexure cracks and compressive failure of concrete in its upper zone. With increasing load, a sudden shear failure occurred close to the column. Subsequently, there was a sudden drop in load to roughly 596 kN.

#### 3.1.3. Strengthened Specimen BS-S1

**Figure 8:**
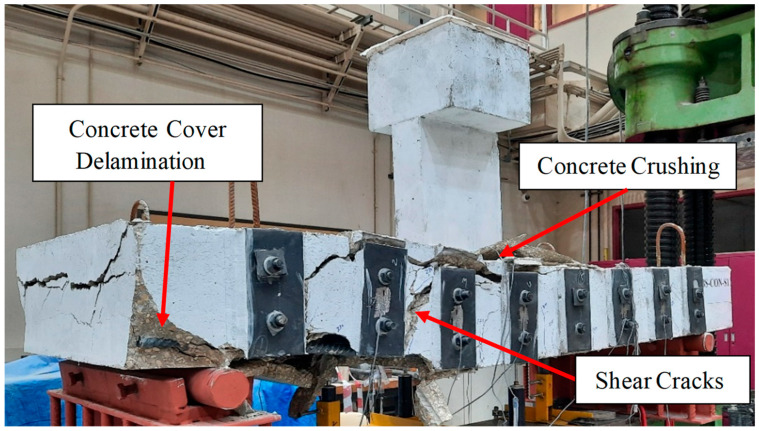
Final mode of specimen BS-S1.

As depicted in Figure 8, the wide beam failed due to diagonal splitting in shear mode. The initial visible crack was observed when the load reached 50 kN. At a load level of about 333 kN, failure of the specimen occurred. Finally, concrete crushing was noted on the upper face of the specimen within the maximum moment zone. As seen from Figure 8, the failure mode of this specimen was comparable to the failure mode noticed in the reference wide beam (BS-CON). The failure mode indicates that the presence of steel plates does not have a significant effect on strengthening RC wide beams with planted columns, which contrasts with the findings of Abbas et al. [3]. These variations can be attributed to the large transverse spacing between the steel plates and the ratio of width (*b*) to effective depth (*d*) of the wide beam; in this study, the ratio (*b*/*d*) was 3.9, whereas in their study, it was 2.4. To elaborate on why this was so critical, the externally bonded steel plates in our specimen were separated by the beam’s full 650 mm width. This large transverse distance means that the plates failed to create a cohesive shear-resisting system across the beam’s cross-section. A diagonal shear crack was able to form and spread through the wide, unplated concrete core between the plates without being effectively stopped. Consequently, the plates acted merely as isolated side stiffeners rather than as an integrated shear reinforcement system. The side steel plates with wide transverse spacing did not cross the internal diagonal cracks, and hence failed to stop their propagation. They provided negligible confinement to the concrete and minimal restraint against the crack widening. This result underscores a key principle: for wide beams with a high width-to-depth ratio like the 3.9 in our study, a strengthening scheme must provide distributed reinforcement across the entire width to be truly effective.

#### 3.1.4. Strengthened Specimen BS-S2

**Figure 9:**
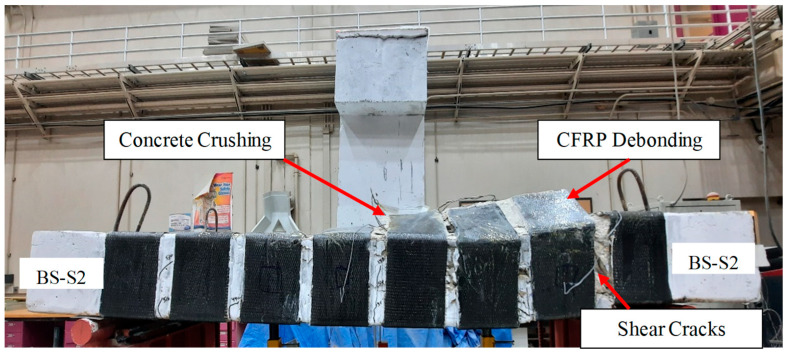
Final mode of specimen BS-S2.

Figure 9 illustrates the mode of failure of beam BS-S2. The first visible flexural crack appeared at a load of 63 kN in the bottom zone of the wide beam. When the load increased, the flexure cracks propagated upward, followed by diagonal shear cracks at 480 kN. The wide beam experienced flexural failure at 600 kN, which is 81% greater than the control beam BS-CON, because of the debonding of the CFRP sheet, as seen in Figure 9. Notably, this technique was chosen to enhance the confinement of the specimen. The success of this hybrid strengthening technique in specimen BS-S2 lies in the synergistic action of its two complementary components. Firstly, the externally bonded CFRP U-wraps provided continuous confinement to the sides and bottom of the beam, acting like an external jacket to effectively control the initiation and growth of diagonal shear cracks. Unlike the discrete steel plates in BS-S1, this continuous jacketing effect maintained the integrity of the concrete core. Secondly, and critically, the planted steel U-stirrups were installed from the top surface in between the CFRP strips. Their primary role was to act as additional, independent shear reinforcement, thereby reducing the effective transverse spacing between the shear-resisting elements. This created a much denser and more uniformly distributed web of reinforcement across the entire width of the beam, thereby crossing the internal diagonal cracks and stopping their propagation. This combined system of external wraps and internal stirrups was far more effective at intercepting and controlling diagonal shear cracks than the widely spaced steel plates in BS-S1. Together, they created a robust shear-resisting mechanism that successfully carried the load capacity and shifted to a more ductile failure mode.

### 3.2. Load Versus Displacement and Strain Response

**Figure 10:**
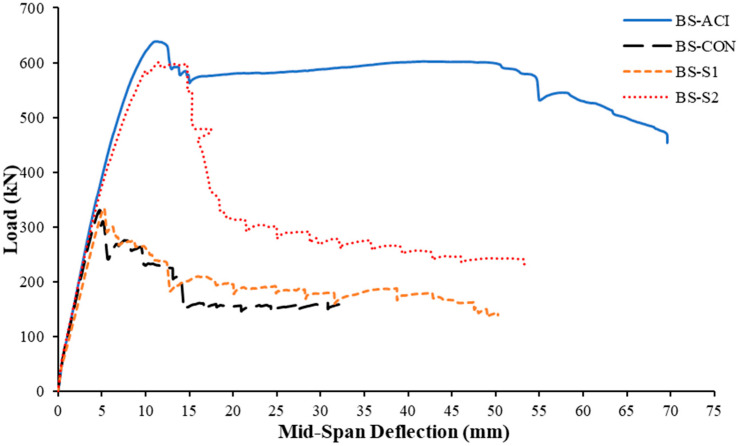
Load–displacement curves of test specimens.

This section discusses the characteristics of load–displacement variation for the four test specimens. Figure 10 illustrates the load–displacement variation for all configurations. As seen in Figure 10, the response of the control specimen (BS-CON) closely aligned with that of the steel plate strengthened specimen (BS-S1), indicating nearly identical structural performance under loading. The initial slope of the load-deflection curve represents the uncracked stiffness (*k_un_*), while the second slope corresponds to the effective post-cracking stiffness (*k_post_*). The third slope of the curve indicates the secant stiffness (*k_s_*). As illustrated in Figure 10, the uncracked stiffness of the strengthened wide beam specimens closely matched that of the reference specimen (BS-ACI). The experimental results, which include the load–displacement response and the corresponding stiffness parameters, are summarized in Table 3 and Table 4, respectively. Experimental strains are given in Table 5. Table 4 presents the calculated energy dissipation (*E*_uf_), which was estimated as the area under the load–displacement curve to failure, and up to the ultimate state as *E_us_*. In this study, the ultimate state was identified by a reduction of approximately 25% in the peak load capacity [30]. Displacement ductility was evaluated as an indicator of the deformation capacity of the beams. It was defined as the ratio of the deflection at the peak load ($\Delta_{pu}$) to the yield deflection ($\Delta_{y}$), expressed as $\mu_{\Delta }=\Delta_{pu}/\Delta_{y}$ [31]. In addition to this conventional definition, two alternative measures were considered. Firstly, $\mu_{\Delta ,75\%}$ was calculated as the ratio of the deflection corresponding to 75% of the ultimate state load on the descending branch of the curve to the yield deflection. The second displacement ductility was corresponding to the concrete crushing ($\mu_{\Delta ,cr}$), which was estimated as the ratio of the deflection measured at the onset of concrete crushing to the yield deflection, thereby reflecting the ductility capacity up to material failure. All displacement ductility values are presented in Table 4.

#### 3.2.1. Control Specimen BS-CON

For specimen BS-CON, the load rose almost linearly up to a peak of 330 kN, with corresponding deflection of approximately 4.7 mm (Figure 10). Thereafter, a sudden decrease occurred in the load to about 241 kN. The load then began to gradually decrease, attributable to the shear failure of the specimen, which occurred when the deflection was nearly 33 mm. The maximum strain in the longitudinal rebars in the bottom layer was 0.00189 mm/mm at a load of 330 KN, which did not yield due to shear failure occurring as shown in Figure 10. After this load, the strain in flexure rebars started to decrease.

#### 3.2.2. Control Specimen BS-ACI

**Figure 11:**
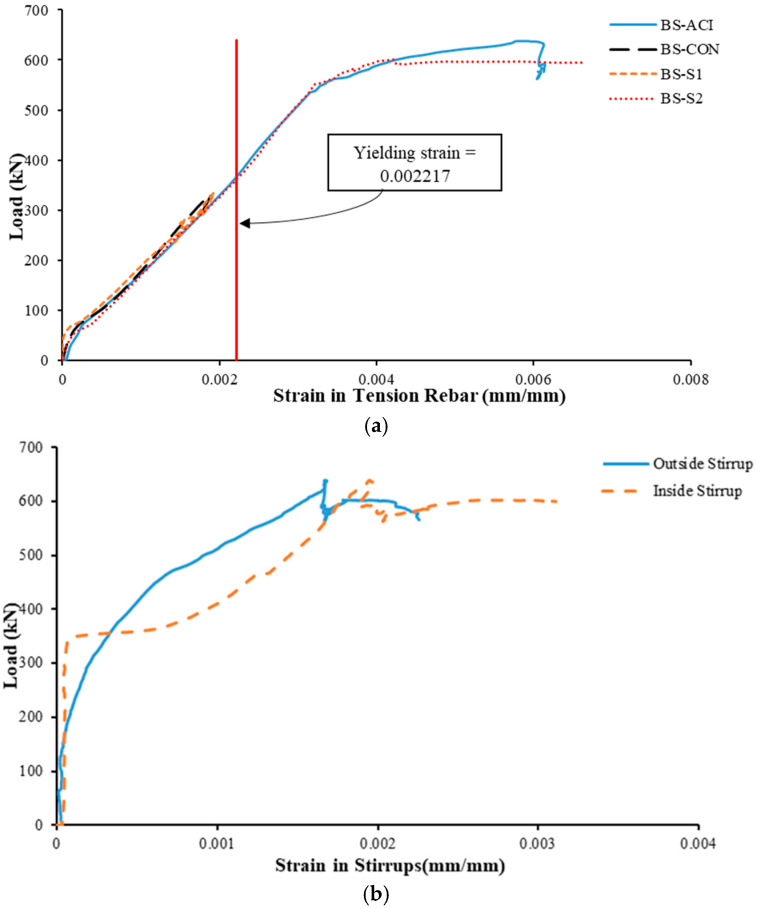

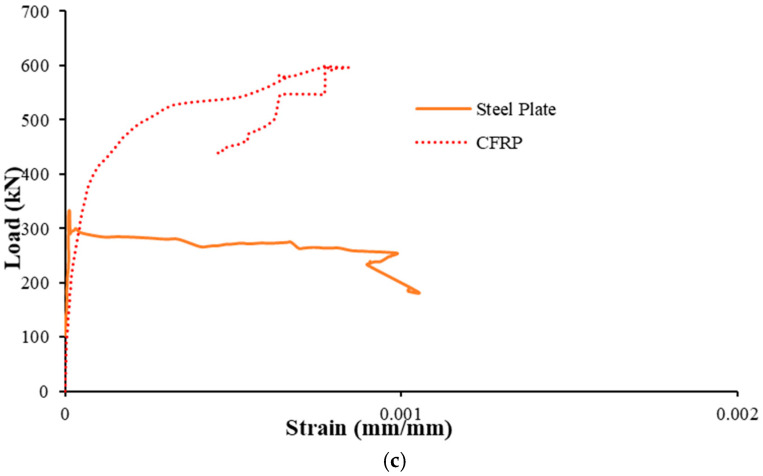
Load versus strain of (**a**) Longitudinal tension rebars; (**b**) Stirrups of specimen BS-ACI; (**c**) Strengthening schemes.

As shown in Figure 10, the BS-ACI wide beam revealed a load-carrying capacity of 638 kN with mid-span deflection of 11 mm. However, the ultimate deflection of the specimen was approximately 70 mm. Once the peak load was attained, it dropped to 577 kN. Following that, a minor increase in load was observed, which continued until it reached 593 kN, when the deflection was nearly 51 mm. Subsequently, the load started to decrease again until the specimen completely failed. Figure 10 clearly showed that the maximum load of this specimen was around double that of the reference specimen BS-CON. The maximum strain in the longitudinal rebars in the bottom layer was 0.00615 mm/mm at a load of 592 kN, as depicted in Figure 11a. Transverse steel rebars experienced different strain levels based on their locations. The internal transverse steel rebars reached the ultimate strain value of 0.003 mm/mm, while the outer transverse steel rebars reached the highest value of 0.00226, at loads of 0.94*P_u_* and 0.89*P_u_*, respectively, as shown in Figure 11b.

#### 3.2.3. Strengthened Specimen BS-S1

As clarified in Table 3 and Figure 10, the adhesively bonded transverse steel plates used in this specimen had a negligible influence on the enhancement in the peak load. The ultimate load reached approximately 334 kN, representing an improvement of only about 1% compared to the control specimen (BS-CON). Upon achieving this peak load, the load abruptly declined to approximately 291 kN. Subsequently, the load continued decreasing to about 183 kN, corresponding to a deflection of about 12.8 mm. Then, the load experienced a slight increase to approximately 209 kN corresponding to a deflection of about 16 mm, after which it gradually decreased until the specimen lost its resistance and shear failure occurred. At the peak load, the main rebars in the bottom layer exhibited a maximum strain of 0.001925 mm/mm, which did not reach the yield strain, as seen from Figure 11a. The maximum strain measured in steel plates was 0.001 mm/mm, as seen from Figure 11c. It is identified that the recorded values of strains at steel plates are significantly less than the yield strain, which means that the plates remained in the elastic stage throughout the test. This was further demonstrated by the visual evaluation of the strengthened scheme following the test.

#### 3.2.4. Strengthened Specimen BS-S2

The hybrid strengthening scheme (CFRP sheet and U-stirrups) used in this specimen (BS-S2) revealed considerable enhancement in peak load compared to the control specimen (BS-CON), as presented in Table 3 and Figure 10. This effect may be partially attributed to the reduction in transverse spacing between the CFRP strips and U-stirrups in the retrofitted wide beam with planted columns. The ultimate load for the specimen (BS-S2) was about 600 kN, which is corresponding to an increase of 82% in comparison with the shear-deficient control beam (BS-CON) at deflection about 11 mm. After attaining the ultimate load, the load dropped to approximately 322 kN, then the decrease continued gradually until failure occurred and the wide beam lost its capacity. The ultimate displacement for this specimen was 53 mm. The rebar’s strain increased until it peaked at its maximum level (0.66%) at a load of 595 kN. Although the CFRP did not rupture (Figure 11c), it did reach a maximum strain of 0.0842% since the shear failure occurred during CFRP debonding and not where the strain gauges were placed.

### 3.3. Comparison of Test Results

**Figure 12:**
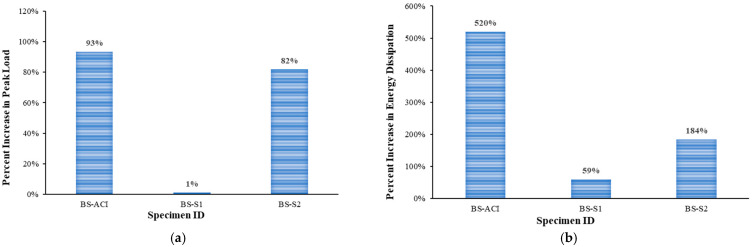
Percentage of increase compared to BS-CON: (**a**) peak load; (**b**) dissipated energy at failure (*E_uf_*).

**Figure 13:**
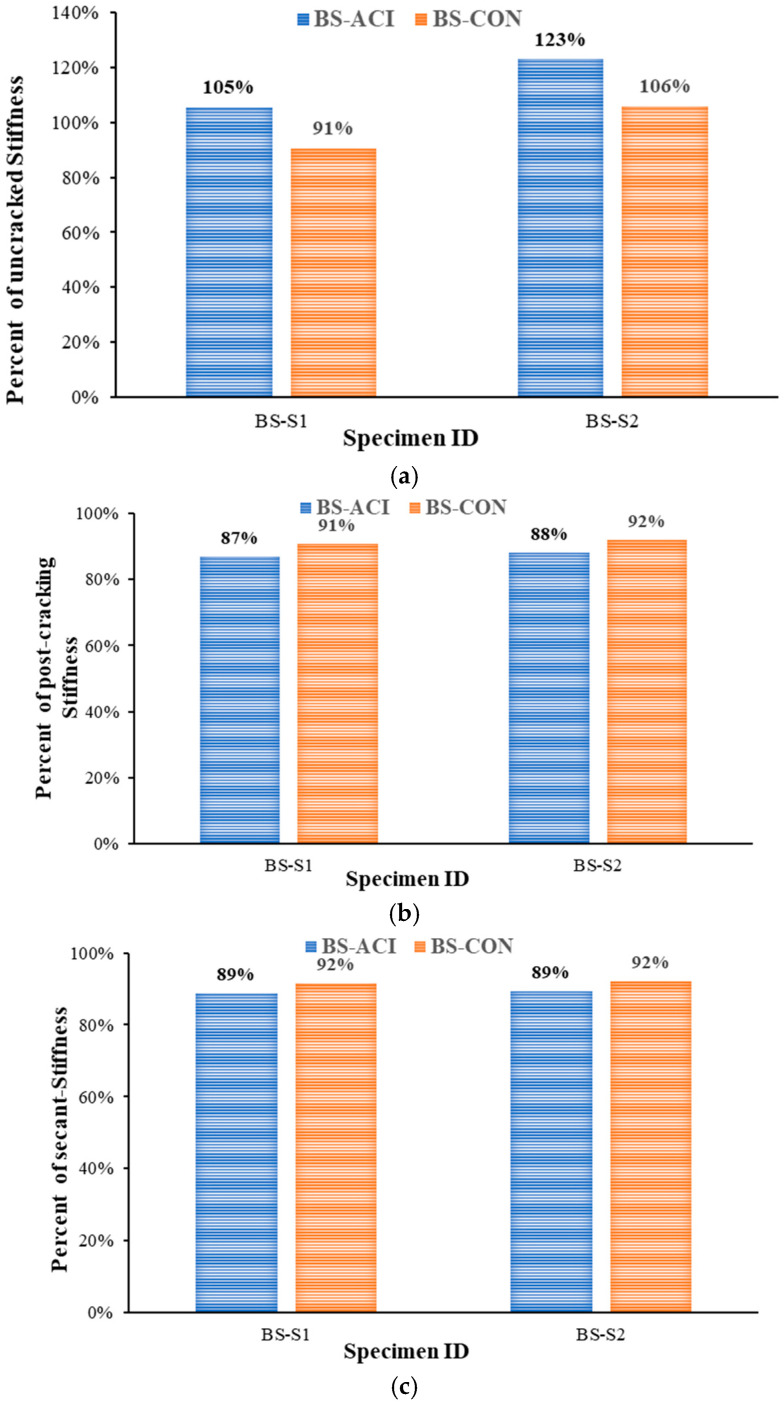
Comparison of stiffness of strengthened specimens with control specimens: (**a**) Uncracked stiffness; (**b**) Post-cracking stiffness; (**c**) Secant stiffness.

Figure 12 illustrates the effect of strengthening schemes on peak load and dissipated energy in comparison with the control beam (BS-CON). However, Figure 13 shows the stiffness of the two strengthened specimens with respect to the BS-CON and BS-ACI. Specimen BS-S1 demonstrated a load-carrying capacity nearly identical to that of the control specimen (BS-CON). This was due to the lack of a significant effect of the steel plate on strengthening the specimen due to the large spacing available between the steel plates. The application of CFRP and U-shaped stirrups for shear strengthening increased the shear resistance of the beam by approximately 82% in relation to the control beam, as shown in Figure 12. CFRP with U-stirrups strengthening scheme (BS-S2) showed an increase in uncrack stiffness by 6% in comparison with control specimens BS-CON, as indicated in Figure 13 and Table 4. However, the secant stiffness was not regained relative to the control specimen (BS-ACI).

The specimen BS-ACI demonstrated a displacement ductility ($\mu_{\Delta }$) 60% larger than specimen BS-CON, indicating an improved deformation capacity. Also, specimen BS-S2 exceeds the control specimen BS-CON by 40%. Additionally, the displacement ductility ($\mu_{\Delta }$) of retrofitted beam BS-S1 was identical to that of BS-CON, showing no improvement in the ductility (Table 4). The ductility, $\mu_{\Delta ,75\%}$, of specimen BS-ACI significantly increased by 350% compared to the control BS-CON. On the other hand, the ductility, $\mu_{\Delta ,75\%}$, of specimens BS-S1 and BS-S2 was similar to that of specimen BS-CON as presented in Table 4. The displacement ductility at concrete crushing ($\mu_{\Delta ,cr}$) improved for specimens BS-ACI and BS-S1 by 41% and 40%, respectively, compared to the specimen BS-CON. In contrast, the displacement ductility ($\mu_{\Delta ,cr}$) of specimen BS-S2 was lower than that of specimen BS-CON by 4%, as listed in Table 4.

## 4. Analytical Prediction of Shear Capacities

### 4.1. Control Specimens

#### 4.1.1. Calculation of Flexural Capacity

**Figure 14:**
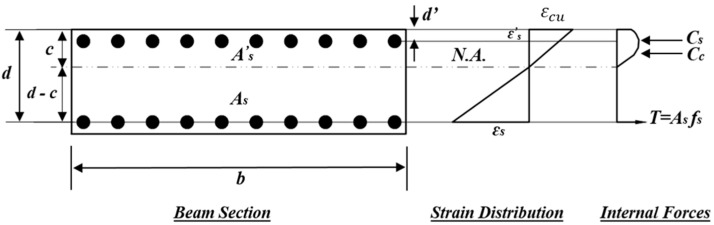
Stress–strain distribution for the wide beam section.

The flexural capacity estimation was required for the design of control specimens. The control specimen, BS-CON, was designed to have a flexural capacity exceeding its shear strength. However, the other control beam, BS-ACI, was designed to have its shear capacity exceed its flexural strength. The stress and strain distribution for the wide beam section is illustrated in Figure 14. Calculation of flexural and shear capacities of control wide beams is detailed below.

**Figure 15:**
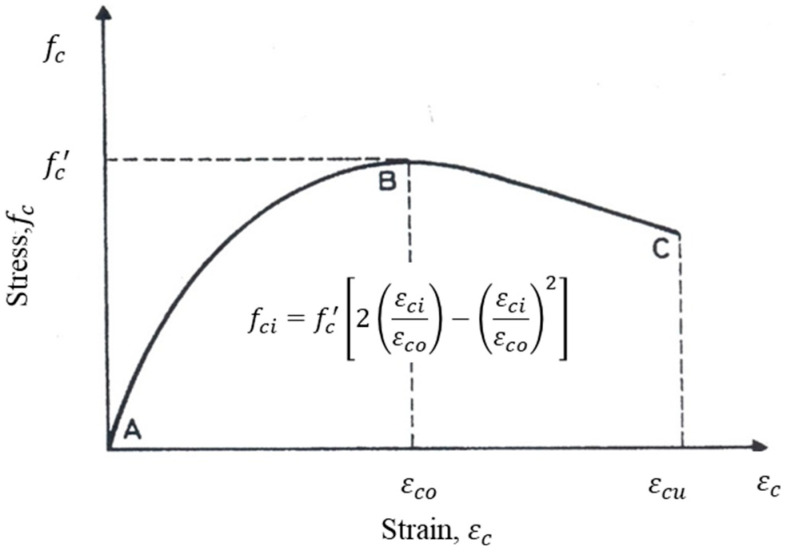
Stress–strain relationship as proposed by Hognestad [32].

To evaluate the moment capacity, the Hognestad model [32] was employed to represent the stress–strain behavior of normal-strength concrete (Figure 15), and expressed as:

(1) $$f_{ci}=f_{c}^{\prime }\left[2\left(\frac{\varepsilon_{ci}}{\varepsilon_{co}}\right)-{\left(\frac{\varepsilon_{ci}}{\varepsilon_{co}}\right)}^{2}\right]$$

where $f_{c}^{\prime }$ denotes the compressive strength of concrete,$\;\varepsilon_{co}$ denotes the concrete strain corresponding to maximum concrete compressive strength, $f_{c}^{\prime }$, and $\varepsilon_{ci}=\left(\frac{c-d_{i}}{c}\right)\times \varepsilon_{cu}$.

By using laminar approach, the concrete compressive force $C_{c}$ at each layer across the depth of the concrete cross-section is given by:

(2) $$C_{ci}=f_{ci}\;b\;h_{i}$$

where $h_{i}$ is the thickness of each layer, and b is the width of wide beam. The total compression force provided by concrete,$\;C_{c},$ is the summation of the compressive forces of all the layers across the depth of the concrete cross-section.

(3) $$C_{c}=\sum_{i=1}^{n}C_{ci}=\sum_{i=1}^{n}f_{ci}\;b\;h_{i}$$

where *n* = the number of layers.

**Figure 16:**
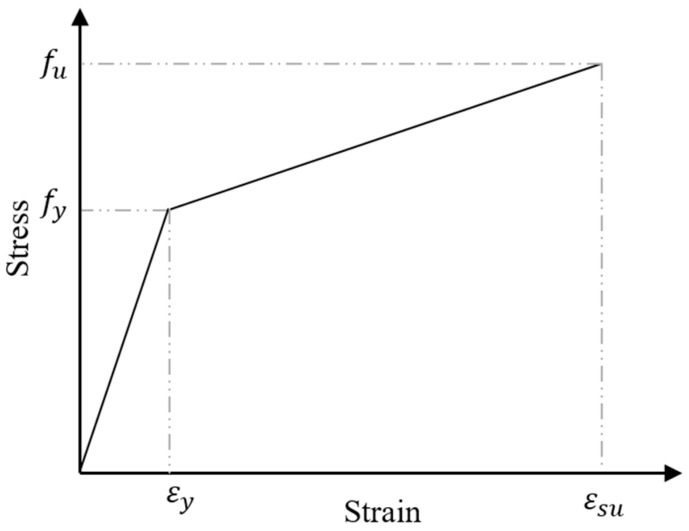
Bilinear stress–strain model for steel reinforcement.

The tensile and compressive strains in the reinforcement bars were determined from the strain variation illustrated in Figure 14. The stresses in the reinforcement were then evaluated based on an assumed bilinear stress–strain relationship, depicted in Figure 16. The tensile and compressive forces in the steel reinforcement were computed using the following expression:

(4) $$T=A_{s}\times \;\;f_{s}$$

(5) $$C_{s}={A_{s}}^{\prime }\times \;{f_{s}}^{\prime }$$

where $A_{s}=$ area of rebars in tension; ${A_{s}}^{\prime }=$ area of rebars in compression; and

(6) $$f_{s}=f_{s}^{\prime }=\left\{\begin{array}{c} \varepsilon_{s}{\;E}_{s}\;\;\;\;\;\;\;\;\;\;\;\;\;\;\;\;\;\;\;\;\;\;\;\;,\;\;{\;\varepsilon }_{s}\leq {\;\varepsilon }_{y}\\ f_{y}+\frac{f_{u}-f_{y}}{{\;\varepsilon }_{su}-{\;\varepsilon }_{y}}({\;\varepsilon }_{s}-{\;\varepsilon }_{y}),\;\;{\;\varepsilon }_{y}<{\;\varepsilon }_{s}<{\;\varepsilon }_{su} \end{array}\right.$$

Therefore, the equation representing section equilibrium is

(7) $$T=C_{c}+C_{s}$$

The depth of neutral axis (*c*) was determined through iteration by satisfying equilibrium.

The flexure capacity was then estimated using the following equation:

(8) $$M_{u}=\sum_{i=1}^{n}C_{ci}\;\left(d-d_{i}\right)+C_{s}\;(d-d^{\prime })$$

where d= beam’s depth up to the centroid of tensile rebars; $d^{\prime }=$ depth up to the centroid of compressive reinforcement.

The peak load was calculated as:

(9) $$P_{u}=\frac{4M_{u}}{L}$$

where L= the span of the wide beam.

In this study, it should be noted that this method was adopted for all tested specimens for calculation of the flexure capacity of wide beams.

#### 4.1.2. Calculation of Shear Capacity

The shear resisting capacity of the control specimen (BS-CON) was calculated from

(10) $$V_{u}=V_{c}+V_{s}$$

where *V_u_* is the ultimate shear strength of the beam’s section, *V_c_* is the shear contribution from concrete, and vs. is the shear contribution from transverse reinforcement. In specimen (BS-CON), the shear resistance offered by stirrups to the overall shear capacity is considered negligible, since a few stirrups were used to hold the longitudinal rebars as previously discussed. Thus, the ultimate shear strength (*V_u_*) is given by:

(11) $$V_{u}=V_{c}$$

The concrete shear capacity ($V_{c}$) can be predicted either using empirical or analytical expressions derived for evaluating the shear resistance of RC wide beams having planted columns as shown in Table 6.

The predictive accuracy of the various models was evaluated against the experimental results of the shear-deficient control specimen, BS-CON, with the predicted shear capacity ($V_{c}$) and the experimental-to-predicted ratio ($V_{c,exp}$/$V_{c}$) included in Table 6. The comparison reveals a significant variation in the predictions. Several models, such as the CSA A23.3-14 [33] and the ACI 318 [5] equations, proved to be overly conservative, with ratios of 1.57 and 1.38, respectively. Conversely, models including Eurocode 2 [34] and Rebeiz [38] were found to be non-conservative, with ratios of 0.94. The JSCE Guidelines [36] provided the most accurate prediction, yielding a measured-to-predicted ratio of 1.04, which indicates a slight and appropriately safe level of conservatism. Therefore, due to its demonstrated reliability for this study’s specific wide beam configuration, the JSCE [36] equation was adopted for calculating the concrete shear contribution ($V_{c}$) in the analytical assessment of all tested specimens. This variation is likely because many of these expressions were developed for conventional beams, whereas the specimens in this study are wide beams with a width-to-depth ratio of 3.9, highlighting the importance of this verification.

The predicted ultimate load of the wide beam was derived using the following analytical expression:

(12) $$P_{u}=2V_{c}$$

It should be noted that the ultimate load, $P_{u}$, of specimen BS-CON is the least of that given by Equations (9) and (12).

For the second control specimen (BS-ACI), the ultimate shear capacity ($V_{u}$) is calculated using:

(13) $$V_{u}=V_{c}+V_{s}$$

The existing codes of practice do not consider the effect of support width, and neither do they incorporate the interaction between the longitudinal and transverse stirrups-leg spacing in their shear resistance prediction equations for wide RC beams. Whereas the equation developed by Alluqmani [41] is designed to take these parameters into account and is expressed as:

(14) $$V_{s,d}=K_{sd}\times V_{s}=\sqrt{\frac{s_{L}}{s_{w}}}\times \sqrt{k_{s}^{\left(1-k_{s}\right)}}\times V_{s}$$

where $k_{s}=\frac{b_{s}}{b_{w}}$. The above equation was used to estimate the shear strength provided by stirrups.

### 4.2. Strengthened Specimens

#### 4.2.1. Estimation of Shear Capacity for BS-S1

For steel plates strengthened specimen (BS-S1), the shear resistance was estimated from:

(15) $$V_{u}=V_{c}+V_{sp}$$

where *V_u_* denotes the ultimate shear strength of beam’s section, *V_c_* represents the shear resistance offered by concrete, and *V_sp_* corresponds to the shear resistance offered by the steel plates. $V_{c}$ was estimated by equations at explained previously in Section 4.1.2.

The most important note is that the steel plates strengthening scheme did not have a significant increase in the shear strength compared with control specimen (BS-CON) since the steel plates system did not experimentally work. The failure of using steel plates system is attributed to the external strengthening with a large transverse spacing (660 mm) between steel plates, which exceeds the limit of transverse spacing as recommended by Egyptian code ECP 203–2018 [42] and Shuraim [12]. Consequently, the steel plates’ shear strength contribution can be ignored. The peak load of the specimen, governed by its shear strength, was calculated based on the following expression

(16) $$P_{u}=2V_{c}$$

#### 4.2.2. Estimation of Shear Capacity for BS-S2

The shear capacity of specimen BS-S2 was computed from:

(17) $$V_{u}=V_{c}+V_{f}{+\;V}_{s2}$$

where $V_{f}$ represents the shear resistance offered by the CFRP sheet,$\;{\;V}_{s2}$ denotes to the shear resistance provided by 10 mm diameter U-stirrups.

As per the ACI 440.2R-17 [29], the shear resistance offered by CFRP shear reinforcement to the total shear capacity was assessed using the following expression:

(18) $$V_{f}=\frac{A_{fv}{\;f}_{fe\;}d_{fv}}{s_{f}}$$

where $A_{fv}$ is the area of cross-section of the CFRP shear reinforcement and is calculated as $A_{fv}$*=*$2nt_{f}w_{f}$, with n denoting the number of CFRP layers, $t_{f}$ is the thickness of a CFRP layer and $w_{f}$ is the width of the CFRP strips. $d_{fv}$ represents the effective depth of CFRP shear reinforcement, $s_{f}$ denotes the center-to-center spacing of CFRP strips, and ${\;f}_{fe\;}$ is the effective stress in the CFRP layers, given by

(19) $${\;f}_{fe\;}={E_{f}\;\varepsilon }_{fe}$$

where $E_{f}$ denotes the tensile elastic modulus of the CFRP layers; and $\varepsilon_{fe}$ represents the effective strain in the CFRP layers. The value of $\varepsilon_{fe}$ is determined using the bond reduction coefficient $\kappa_{v}$, as defined by the following equations:

(20) $$\varepsilon_{fe}=\kappa_{v}\varepsilon_{fu}\leq 0.004$$

(21) $$\kappa_{v}=\frac{k_{1}\;k_{2}\;L_{e}}{11,900\varepsilon_{fu}}\leq 0.75$$

where $k_{1}$ is a modification factor applied to $\kappa_{v}$ to account for concrete strength, $k_{2}$ is a modification factor reflecting the influence of the CFRP wrapping scheme, $L_{e}$ denotes the effective bond length of the CFRP laminate, and $\varepsilon_{fu}$ is the design rupture strain of FRP reinforcement. These parameters are estimated from the following equations

(22) $$k_{1}={\left(\frac{f_{c}^{\prime }}{27}\right)}^{2/3}$$

(23) $$k_{2}=\frac{d_{fv}-L_{e}}{d_{fv}}$$

(24) $$L_{e}=\frac{23,300}{{\left(nt_{f}E_{f}\right)}^{0.58}}$$

(25) $$\varepsilon_{fu}=C_{E}\;{\varepsilon_{fu}}^{*}$$

where $C_{E}$ is the environmental reduction factor (=0.95); ${\varepsilon_{fu}}^{*}$ denotes the ultimate rupture strain of the CFRP reinforcement.

While the ACI 440.2R-17 [29] equations provide the fundamental basis for calculating the shear contribution of FRP systems, they do not explicitly account for the potential reduction in effectiveness caused by large transverse spacing between reinforcement legs, a key characteristic of wide beams. In such members, shear stress distribution can be non-uniform, and not all reinforcement may be fully effective. To address this limitation and provide a more accurate prediction for the tested wide beam, the shear strength provided by both the CFRP strips $\left(V_{f}\right)$ and the planted U-stirrups ($V_{s2}$) was modified using the design factors proposed by Alluqmani [41].

### 4.3. Peak Load Comparison of Tested Specimens

**Figure 17:**
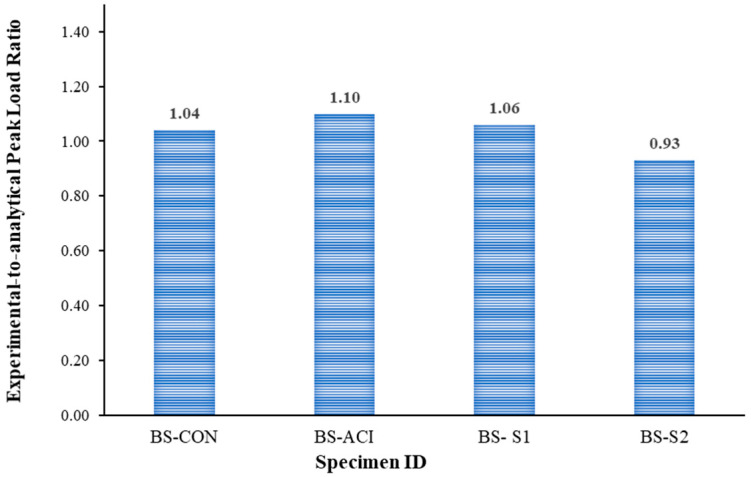
Comparison of predicted ultimate load with experimental load of tested specimens.

Table 7 and Figure 17 illustrated the ultimate loads compression between all tested specimens in this study. The results showed a good match between experimental and analytical peak load calculations with prediction errors of no more than 10%.

## 5. Conclusions

The major findings drawn from this investigation are as follows:

- Shear reinforcement in RC wide beams, provided in accordance with the design codes (e.g., ACI 318-19), changes the mode of failure from shear to flexure, thereby making it ductile.
- For avoiding brittle failure in RC wide beams supporting columns, their shear strength must exceed flexural capacity, by designing as per the current code requirements.
- The failure mode observed in the strengthened specimen BS-S1 indicates that the presence of the external steel plates technique had an insignificant influence on the shear resistance of wide beams compared to the control specimen BS-CON, which is deficient in shear. Therefore, it is suggested to add internal stirrups along with external plates.
- The shear strengthening of CFRP U-wraps combined with steel U-stirrups in the strengthened specimen BS-S2 improved the shear resistance of wide beams by 82% compared to the control specimen. Moreover, this strengthening scheme showed an increase in uncrack stiffness by 6% in comparison with the control specimens BS-CON.
- The strengthening schemes applied to specimens BS-S1 and BS-S2 led to increases in dissipated energy of approximately 59% and 184%, respectively, in comparison with the control specimen (BS-CON).
- It is recommended to review and incorporate international design codes that address the specific challenges of wide beams supporting columns to ensure adherence to best practices in structural safety. Also, it is recommended to conduct a finite element analysis to model the shear response of RC wide beams having columns and compare the results with the experimental findings obtained in this investigation.

## Figures and Tables

**Table 1:** Details of test specimens.

Specimen ID	Shear Stirrups	Specimen Type	Shear Strengthening	No. of Beams
BS-CON	2L-Ø6@500	Control specimen	None	1
BS-ACI	6L-Ø8@80	Control specimen	None	1
BS-S1	2L-Ø6@500	Strengthened specimen	Scheme 1—Externally bonded steel plates	1
BS-S2	2L-Ø6@500	Strengthened specimen	Scheme 2—Externally bonded CFRP U-wraps combined with planted steel U-stirrups	1
Total No. of specimens				4

**Table 2:** Material Properties.

**Concrete**		
28-day compressive strength	39 MPa	
Compressive strength on testing day	40 MPa	
**Steel reinforcement**		
**Rebar diameter (mm)**	**Tensile strength (MPa)**	
	**Yield**	**Ultimate**
Ø 6	440	505
Ø 8	635	667
Ø 10	547	572
Ø 18	526	654
**CFRP Sheet**		
Elastic modulus	71.46 GPa	
Rupture strain	0.01 mm/mm	
Ultimate tensile strength	710 MPa	
Thickness per layer	1.3 mm	
**Steel plate**		
Thickness	10 mm	
Elastic modulus	200 GPa	
Yield tensile strength	249 MPa	
Ultimate tensile strength	335 MPa	
**Fast Curing Anchoring Adhesive (Sika AnchorFix^®^-2)**		
Compressive strength	70 MPa	
Tensile strength	29 MPa	
Elastic modulus	3800 MPa	
**Epoxy-based mortar (Sikadur-31)**		
Compressive strength	40–45 MPa	
Tensile strength	15–20 MPa	
Young’s modulus	4.3 GPa	

**Table 3:** Parameters Estimated from the Test Results of Specimens ^a^.

Specimen ID	*P_cr_* (kN)	*P_y_* (kN)	*P_u_* (kN)	Δ*_cr_* (mm)	Δ*_y_* (mm)	Δ*_pu_* (mm)	Δ*_u_* (mm)	Failure Pattern
BS-CON	45	-	330	0.38	-	4.76	9.7	Shear
BS-ACI	74	518	638	0.74	7.25	11.40	68.0	Flexure
BS-S1	51	-	334	0.47	-	5.26	10.8	Shear
BS-S2	63	524	601	0.5	8.20	11.52	16.0	Flexure-shear

^a^ *P_cr_* = cracking load; *P_y_* = yielding load; *P_u_* = peak load; Δ*_cr_* = mid-span deflection at cracking load; Δ*_y_* = mid-span deflection at yield load; Δ*_pu_* = mid-span deflection at peak load; Δ*_u_* = mid-span deflection at ultimate state.

**Table 4:** Parameters for tested specimens ^b^.

Specimen ID	$k_{un}$ (kN/mm)	$k_{post}$ (kN/mm)	$k_{s}$ (kN/mm)	*E_uf_* (kN/mm)	*E_us_* (kN/mm)	$\mu_{\Delta }$	$\mu_{\Delta ,75\%}$	$\mu_{\Delta ,cr}$
BS-CON	119.53	65.02	69.37	6102	2209	1	2	6.8
BS-ACI	102.78	67.99	71.45	37811	37313	1.6	9	9.6
BS-S1	108.30	59.10	63.50	9725	2466	1	2	9.5
BS-S2	126.44	59.84	63.90	17313	6996	1.4	2	6.5

^b^ $k_{un}$ = *P_cr_*/Δ*_cr_*; for BS-CON and BS-S1 $k_{post}$ = (*P_u_* − *P_cr_*)/(Δ*_pu_* − Δ*_cr_*); for BS-ACI and BS-S2 $k_{post}$ = (*P_y_* − *P_cr_*)/(Δ*_y_* − Δ*_cr_*);$k_{s}$ = *P_y_*/Δ*_y_*; *E_uf_* = energy dissipated at failure; *E_uf_* = energy dissipated at ultimate state.

**Table 5:** Experimental results of strain obtained from load-strain curves *.

Specimen ID	ε_s,pu_	ε_su_	ε_sp,u_	ε_s,CFRPu_
BS-CON	0.001884	0.00189	-	-
BS-ACI	0.00592	0.00615	-	-
BS-S1	0.001925	0.001925	0.001	-
BS-S2	0.004179	0.006627	-	0.000842

* *ε_s,pu_*: strain corresponding to ultimate load; *ε_su_*: ultimate steel strain; *ε_sp,u_*: ultimate steel plate strain; *ε_s,CFRPu_*: ultimate CFRP strain.

**Table 6:** Predictive Equations for Shear Strength of Concrete Beams.

Reference	Shear Capacity of Concrete, *V_c_* (Units: N, mm)	*V_c_* (kN)	*V*_c,exp_/*V*_c_
ACI 318 [5]	$V_{c}=\frac{\sqrt{f_{c}^{\prime }}b_{w}d}{6}$ $\mathrm{where}\;b_{w}$ and d are the width and effective depth of the beam	120	1.38
ACI 318 [5]	$V_{c}=\left(0.16\lambda \sqrt{f_{c}^{\prime }}+17\rho_{w}\frac{V_{u}d}{M_{u}}\right)b_{w}d\leq 0.29\lambda \sqrt{f_{c}^{\prime }}b_{w}d$ $f_{c}^{\prime }$ = concrete’s compressive strength $M_{u}$$\;\mathrm{and}\;V_{u}$ = factored bending moment and shear force $\rho_{w}$ = main rebar ratio	120	1.38
CSA A23.3-14 [33]	$V_{c}=\lambda \beta \sqrt{f_{c}^{\prime }}bd_{v}$ λ = factor for concrete unit weight = 1.0 for normal weight concrete $\beta =\frac{0.40}{\left(1+1500\varepsilon_{x}\right)}\times \frac{1300}{\left(1000+s_{ze}\right)}$ where $\varepsilon_{x}=\frac{M_{f}/d_{v}+V_{f}-V_{\rho }+0.5N_{f}-A_{\rho }A_{\rho o}}{2(E_{s}A_{s}+E_{\rho }A_{\rho })}\leq 0.003$ $s_{ze}=\frac{35d_{v}}{15+a_{g}}\geq 0.85d_{v}$ $M_{f}$$\mathrm{and}\;V_{f}$ = factored moment and shear force, where $M_{f}\geq (V_{f}-V_{\rho })$ $d_{v}$ = Effective shear depth, defined as the greater of 0.9 d or 0.72 h where h is the total depth of the beam. $E_{s}$ = modulus of elasticity of steel; $A_{s}$ = Cross-sectional area of the tensile reinforcement. $a_{g}$ = maximum size of coarse aggregate. $f_{c}^{\prime }$ not exceeding 64 MPa	105	1.57
Eurocode 2 [34]	$V_{c}=0.18k\sqrt[{3}]{100\rho f_{ck}}bd\geq 0.35\sqrt{k^{3}f_{ck}}bd$ $k=1+\sqrt{\frac{200}{d}}\leq 2;\rho \leq 0.02$ $f_{ck}\leq 90Mpa$ $f_{ck}=$ Characteristic compressive cylinder strength of concrete	175	0.94
AS 3600 [35]	$V_{c}=\beta_{1}\beta_{2}\beta_{3}bdf_{cv}\sqrt[{3}]{\rho }$ $\beta_{1}=1.1\left(1.6-\frac{d}{1000}\right)\geq 0.8;$$\beta_{2}=1+\frac{N}{14A_{g}}$ for members subjected to axial compression; N = compressive axial force; $1\leq \beta_{3}=\frac{2}{a/d}\leq 2$$;\;f_{cv}=\sqrt[{3}]{f_{ck}}\leq 4Mpa$	172	0.96
JSCE Guidelines [36]	$V_{c}=\beta_{d}\beta_{\rho }f_{vcd}bd$ $\beta_{d}=\sqrt[{4}]{1000/d}\leq 1.5;$ $\beta_{\rho }=\sqrt[{3}]{100\rho }\leq 1.5;$ $f_{vcd}=0.2\sqrt[{3}]{f_{ck}}\leq 0.72$ MPa	158	1.04
Kim and Park [37]	$V_{c}=3.5f_{c}^{\prime \alpha /3}\rho^{3/8}\left(0.4+d/a\right)\left(\frac{1}{\sqrt{1+0.008d}}+0.18\right)bd$ $\alpha =1for\frac{a}{d}\geq 3;$ $\alpha =2-\frac{\frac{a}{d}}{3}for1\leq a/d<3$	171	0.96
Rebeiz [38]	$V_{c}=\left(0.4+\sqrt{f_{c}^{\prime }\rho \left(\frac{d}{a}\right)}\left[10-3A_{d}\right]\right)bd;$ $A_{d}=\left\{\begin{array}{l} \frac{a}{d},\;\;\;1<\frac{a}{d}<2.5\\ 2.5,\;\;\frac{a}{d}\geq 2.5 \end{array}\right.$	175	0.94
Bentz [39]	$V_{c}=\frac{200}{1000+s_{e}}\sqrt{f_{c}^{\prime }}bd$ $\;;s_{e}=\frac{31.5d}{a_{g}+16}$	121	1.36
Cladera and Mari [40]	$V_{c}=0.225\xi \sqrt{100\rho }f_{c}^{\prime 0.2}bd$ $\xi =1+\sqrt{\frac{200}{0.9d}}\leq 2.75;$ $\rho =tensile\;rebar\;ratio\leq 0.02(1+\frac{f_{c}^{\prime }}{100})$	171	0.96

**Table 7:** Peak loads comparisons analytically and experimentally for tested specimens *.

Specimen ID	*P_u,exp_* (kN)	*P_u_* (kN)	*P_u,exp_*/*P_u_*
BS-CON	330	316	1.04
BS-ACI	638	582	1.10
BS-S1	334	316	1.06
BS-S2	601	643	0.93

* *P_u,exp_* is the experimentally obtained ultimate load; *P_u_* is the analytically estimated ultimate load.

## Data Availability

The original contributions presented in this study are included in the article. Further inquiries can be directed to the corresponding author.
